# A Han Chinese Family With Early-Onset Parkinson's Disease Carrying Novel Frameshift Mutation and Compound Heterozygous Mutation of *PRKN* Appearing Incompatible With MDS Clinical Diagnostic Criteria

**DOI:** 10.3389/fneur.2020.582323

**Published:** 2020-10-09

**Authors:** Chenyu Gao, Ting Huang, Rui Chen, Zhenhua Yuan, Youyong Tian, Yingdong Zhang

**Affiliations:** Department of Neurology, Nanjing First Hospital, Nanjing Medical University, Nanjing, China

**Keywords:** Parkinson's disease, early-onset, PRKN mutations, genetic analysis, whole-exome sequence

## Abstract

Around 15% of patients with Parkinson's disease (PD) have a family history, and 5–10% have confirmed genetic causes. *PRKN* is the most common gene responsible for early-onset Parkinson's disease (EOPD), while rare variants of *PLA2G6* likely raise PD susceptibility in the Chinese population. We investigated the genetic information of 13 members of a Han Chinese family with known EOPD by whole-exome sequencing and Sanger sequencing, and analyzed the clinical history, physical examination, blood laboratory test, and brain imaging data of the patients. Two members, including the proband, were suspected of having EOPD. A novel homozygous frameshift mutation, c.856delT, and a compound heterozygous mutation, c.1321T>C/c.856delT of *PRKN*, were identified, as well as two single nucleotide variants of *PLA2G6* and *TENM4*. The proband exhibited a rare symmetrical resting tremor limited to her lower limbs and never exhibited signs of rigidity. ^18^F-DOPA PET/CT scan indicated a symmetrical reduced signaling in the striatum. The novel frameshift mutation and compound heterozygous mutation of *PRKN* are likely to be the genetic causes of EOPD in this family.

## Introduction

Parkinson's disease (PD), the second most common neurodegenerative disorder after Alzheimer's disease (1), affects 1% of persons over the age of 65 and 3% of those older than 80 (2). PD is a type of alpha synucleinopathy with aggregation of abnormally folded α-synuclein proteins, which form intracellular inclusions within Lewy bodies and Lewy neurites, while the specific subnuclei of the substantia nigra pars compacta (SNpc) are damaged, causing the loss of dopaminergic neurons (3). In this way, PD generates a series of clinical symptoms, classified as motor symptoms and nonmotor symptoms. Resting tremor, bradykinesia, and rigidity are defined as classical parkinsonian motor symptoms, which usually develop unilaterally and later spread to the contralateral side, but ordinarily remain asymmetrical throughout the disease course (4, 5). Nonmotor symptoms include hyposmia, cognitive disorder, sleep disorder, autonomic dysfunction, and psychiatric symptoms (4, 5). To diagnose PD, not only are the mandatory criteria needed but also diagnostic exclusion criteria and red flags for PD diagnosis should be considered (4, 6).

Although PD was categorized as an idiopathic disorder due to its unknown etiology, around 15% of the patients have a family history of the disease, and 5–10% have a monogenic form of the disease with Mendelian inheritance (7, 8). At least 23 loci and 19 genes (10 autosomal dominant genes and 9 autosomal recessive genes) for parkinsonism have been identified as pathogenic up to 2018, 10 genes of which are proven to be associated with early-onset Parkinson's disease (EOPD) (9). *PRKN* is the most common gene responsible for EOPD, causing nearly 49% of EOPD cases with autosomal recessive inheritance and 19% of sporadic EOPD cases (10). *PLA2G6* mutations were first reported to be related to adult-onset levodopa-responsive dystonia-parkinsonism in 2009 (11), and more than 18 *PLA2G6* variants have been confirmed to be associated with familial dystonia-parkinsonism and PD thus far (9). It has been suggested that rare variants of *PLA2G6* may raise PD susceptibility in the Chinese population (12).

In this study, we discuss a Chinese family carrying a novel frameshift mutation and a compound heterozygous mutation of *PRKN*, as well as two single nucleotide variants (SNVs) of *PLA2G6* and *TENM4*. We collected the clinical data of the proband as well as the other affected member of the family, and analyzed the family pedigree by investigating the DNA sequencing results of 13 members from four generations. The proband exhibited a rare symmetrical resting tremor limited to her lower limbs, without the appearance of rigidity, which is incompatible with the MDS clinical diagnostic criteria.

## Materials and Methods

### Subjects

We investigated the genetic information of 13 members in a family with EOPD from the Jiangxi Province of Southern China. Two female members appeared to have symptoms of PD, while other family members did not exhibit related clinical manifestations. The two affected family members were examined by three experienced neurologists from Nanjing First Hospital, Nanjing Medical University, and data collected included clinical history review, physical examinations, and blood laboratory tests. All participants, or their legal guardians, provided informed consent. The study was approved by the ethics committee of Nanjing First Hospital, Nanjing Medical University. All procedures were conducted strictly according to the Administrative Regulations on Medical Institutions, issued by the State Council of the P.R. China.

### Genetic Analysis

Peripheral blood samples were collected from 13 family participants for DNA sequencing, including two patients and 11 persons who appeared healthy. Genomic DNA was extracted following the standard protocol of the QIAamp DNA Blood Mini Kit (Qiagen, Hilden, Germany). Exomes of gDNA isolated from the two patients were captured by liquid chip technology. Samples of the two patients underwent paired-end sequencing on the Illumina HiSeq X-ten platform (Illumina Inc., San Diego, CA, USA). Sanger sequencing was performed to validate candidate variants of 13 family participants using an ABI 3730 XL genetic analyzer (Applied Biosystems Inc., Foster City, CA, USA). All potential variants were filtered according to the Single Nucleotide Polymorphism Database, the 1,000 Genomes Project (1000G), and the Genome Aggregation Database (gnomAD). Sorting Intolerant from Tolerant (SIFT) and Polymorphism Phenotyping v2 (Polyphen-2) were used to predict the impact of an amino acid substitution caused by missense mutation on the protein's structure and function. The impact of synonymous variants, short InDel variants, and variants across the intron–exon border was predicted by MutationTaster.

### Neuroimaging Analysis

Computed tomography (CT) and 3.0T magnetic resonance imaging (MRI) scans were performed on the proband's head. She also underwent positron emission computed tomography (PET/CT) scan of the brain, and to eliminate interference, she was injected with imaging agent ^18^F-FDG 24 h after the ^18^F-DOPA injection after a 6-h fast. After 90 min, a static brain scan was performed in accordance with the standard operating procedures of Nanjing First Hospital, and the images were reconstructed with CT-based attenuation correction. The other patient claimed to have difficulty traveling, and thus she was unable to finish the imaging examination.

## Results

### Family Pedigree Map

**Figure 1:**
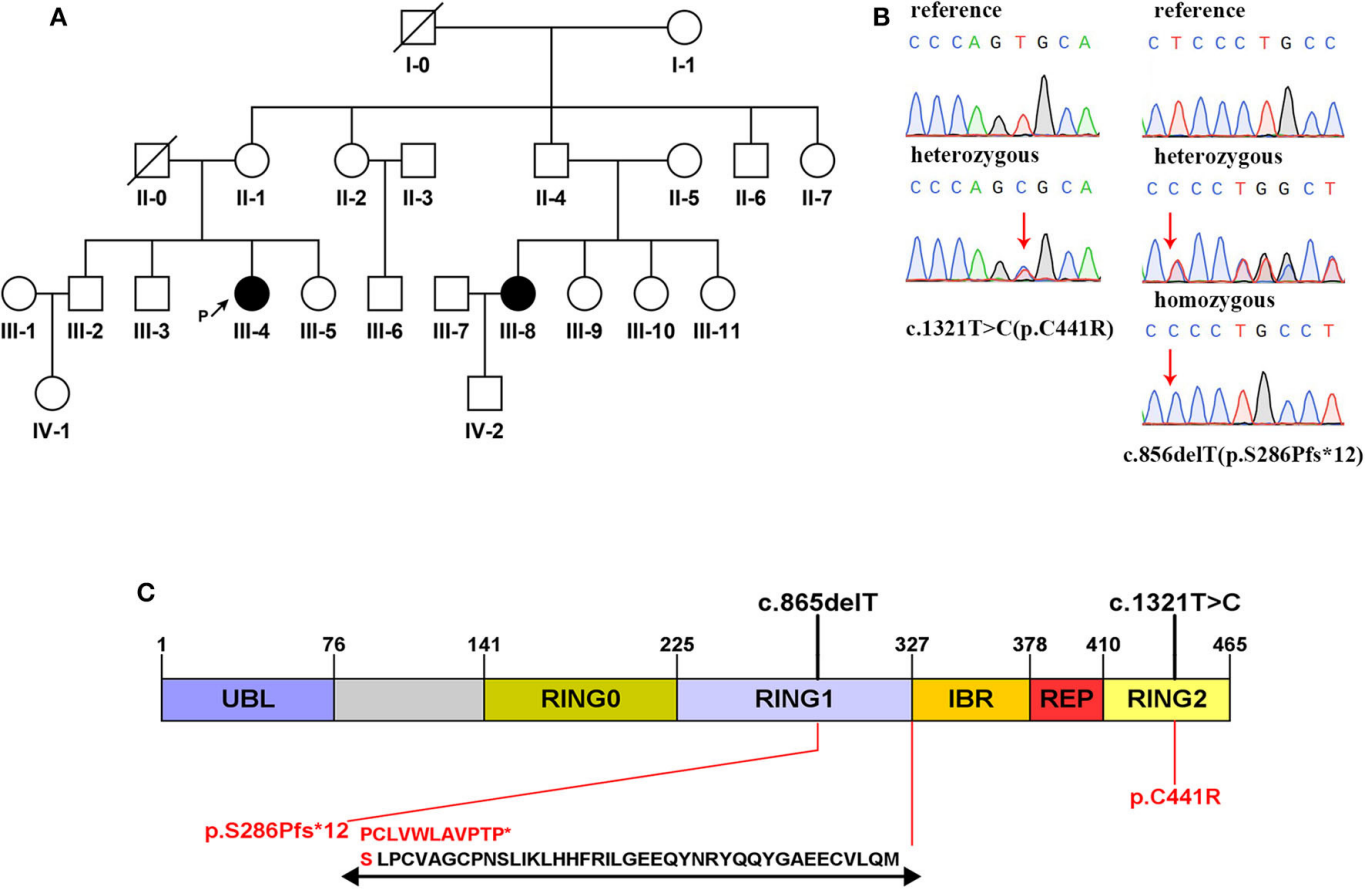
**(A)** Family pedigree map. Circle: female; square: male; black: the affected; white: the healthy; arrow with letter P on the side: the proband; slash: the deceased. **(B)** Sanger sequencing analysis of the mutation c.1321C>T and the mutation c.856delT. **(C)** Domain structure of the parkin protein (13). Two mutations detected in this study are marked in black above the icon. The amino acid changes are marked in red below the icon. Specific amino acid sequence changes caused by the frameshift mutation are shown at the bottom.

A novel frameshift mutation and a compound heterozygous mutation of *PRKN* were detected in a Han Chinese family with a history of EOPD, whose members also carried other SNVs of *PLA2G6* and *TENM4*. The family pedigree was established based on genetic analysis of 13 members from four generations, including 11 unaffected members (Figure 1A, II-1,2,5,6,7, III-2,3,5,6,9, and IV-1) and two with EOPD (Figure 1A, III-4,8). Three members of the family (Figure 1A, I-1, II-4, and IV-2) refused to participate in the study, and another five family members (Figure 1A, II-3, III-1,7,10,11) were not able to take part in the research. There is no consanguineous marriage in the family according to the proband's statement.

### Clinical Features

The proband (III-4) is a 45-year-old woman with an 11-year history of PD-like tremor in both lower limbs. She was found to have resting tremor in two legs at the age of 34 with fatigue complaints. The symptoms gradually worsened in the following years, but still progressed at a slow rate. In 2017, a large-scale polyclinic suspected her of having PD, and she began oral medication (Madopar 125 mg tid) then. When the prescription worked well, the patient reduced the drug dose, which made the symptoms worse again. Routine and biochemical blood examination showed no significant abnormalities. Blood copper, ceruloplasmin, and liver ultrasound were normal, and no K–F rings were observed. Nevertheless, her younger cousin (III-8), another affected member of the family, appears to have typical PD symptoms, including resting tremor, rigidity, and bradykinesia. She first noticed weakness in her left leg and difficulty moving in 2005, without tremor signs at that time. In her left limb, however, she gradually developed tremor, rigidity, and bradykinesia. The symptoms peaked in 2015. She had great difficulty in drinking and dressing, and started to take Madopar at that time. Although the medicine was effective, the patient took it irregularly (125 mg tid/bid). The clinical features of these two patients are summarized in Table 1. One (III-6) of the proband's cousins has a history of psychosis.

**Table 1:** Clinical features of the two patients in this family.

	**Patient ID**	
	**III-4**	**III-8**
Gender	Female	Female
Origin	Han Chinese	Han Chinese
Age at present (years)	45	40
Age at disease onset (years)	34	25
Disease duration (years)	11	15
Body weight (kg)	44	53.8
Body mass index (kg/m^2^)	19.3	21.6
Rest tremor	+	+
Bradykinesia	+^1^	+
Rigidity	–	+
Gait disturbance	–	–
Postural stability	–	–
Asymmetry at onset	–	+
Autonomic dysfunction^2^	–	+
RBD	–	–
Olfactory dysfunction	–	–
Dystonia	–	–
Numbness	–	+
Hallucination	–	–
Hypomnesia	+	+
Clinical response to levodopa	+	+
LEDD (mg/day)	300	200/300^3^
Levodopa-induced dyskinesia	–	–
Wearing-off	–	+
Pack-years of smoking	3.9	1.5–2
Green tea intake	Never	Quit
Other history	None	Appendectomy
		Head and knee trauma
Hoehn and Yahr	2	2.5
MDS-UPDRS score	18 off	80 off
MoCA score	23	26
MMSE score	30	30
HAMD score	11	12
HAMA score	11	15
Phenotypes incompatible with the MDS criteria	Symmetrical parkinsonism;	/
	Parkinsonism limited to lowers limbs for over 3 years	

*RBD, rapid eye movement sleep behavior disorder; LEDD, levodopa equivalent daily dosage; MDS-UPDRS, Movement Disorder Society-Sponsored Revision of the Unified Parkinson's Disease Rating Scale; MoCA, Montreal Cognitive Assessment; MMSE, Mini-Mental State Examination; HAMD, Hamilton Depression Scale; HAMA, Hamilton Anxiety Scale*.

1 *The proband showed a slight slowing of movements in her lower limbs, and the movements of the upper limbs did not slow down significantly*.

2 *The positive autonomic dysfunction features of III-2 include constipation and nightmares*.

3 *The patient takes medication irregularly, taking Madopar 125 mg twice a day or thrice a day*.

### Neuroimaging

**Figure 2:**
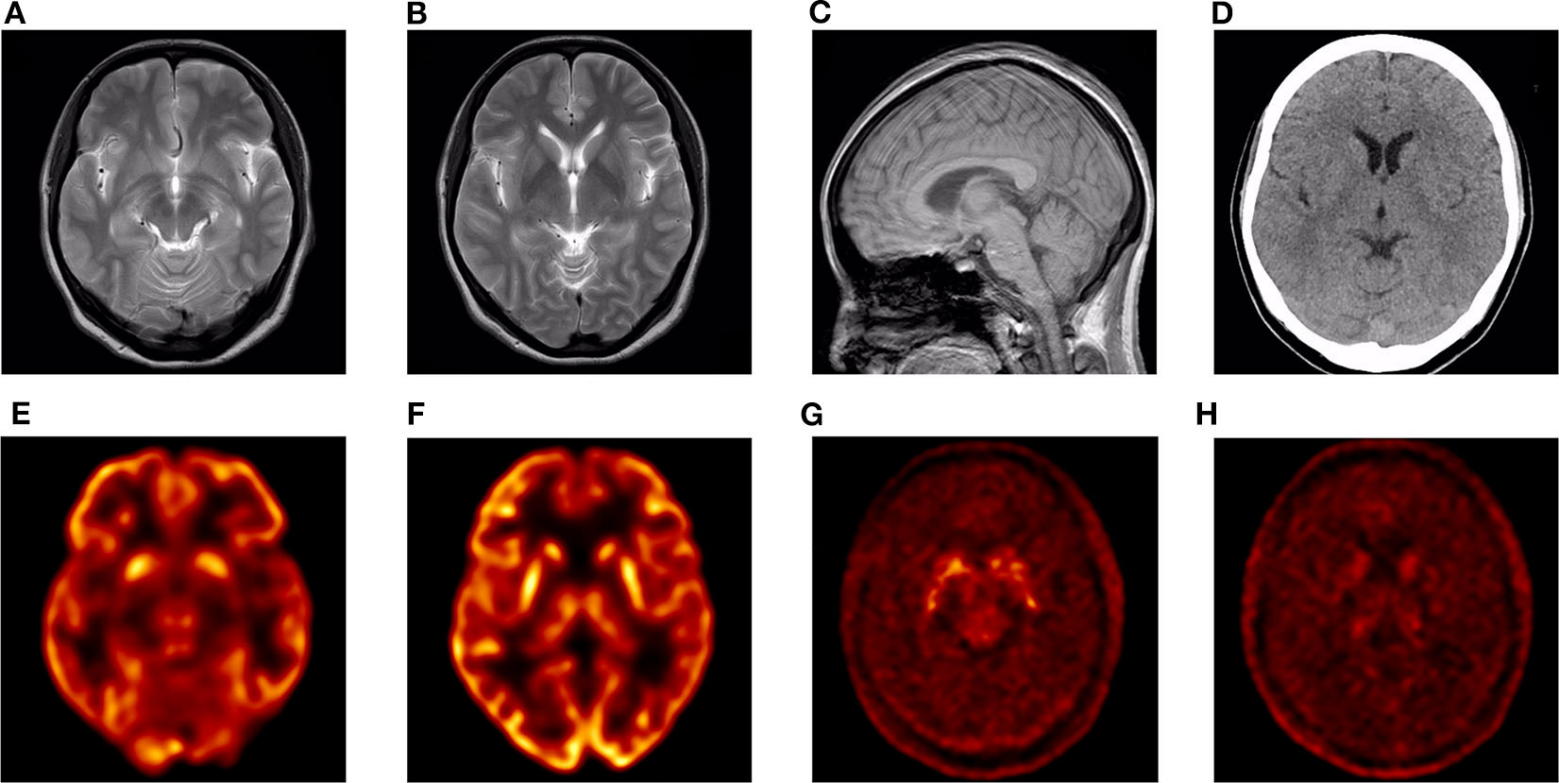
Neuroimaging images of the proband. **(A–C)** 3.0T MRI images: no obvious abnormalities. **(D)** CT image: no obvious abnormalities. **(E,F)** ^18^F-FDG PET/CT image: no obvious abnormalities. **(G,H)** ^18^F-DOPA PET/CT image: bilateral striatum dopamine neurons damaged.

CT and 3.0T MRI scans of the proband were negative, and no obvious brainstem or cerebellar atrophy was observed (Figures 2A–D). The brain PET/CT scan showed some abnormalities. Nothing abnormal was found in her ^18^F-FDG PET/CT imaging, as no reduced signaling was detected in the lenticular nucleus and thalamus (Figures 2E,F). However, ^18^F-DOPA PET/CT scan indicated reduced signaling in the striatum, which was mostly symmetrical (Figures 2G,H).

### Molecular Genetic Findings

Mutations in exon 7 and exon 12 of *PRKN* were detected in the patients by whole-exome sequencing, which reached an average coverage of >100 × and a minimal coverage of 20 × in 97% of the target regions. A compound heterozygous mutation, c.1321T>C (p.C441R)/c.856delT (p.S286Pfs^*^12), was detected in the proband (III-4), and a homozygous mutation, c.856delT (p.S286Pfs^*^12), was detected in her younger cousin (III-8). The two candidate mutations were validated by Sanger sequencing in all participants (Figure 1B). The proband's mother (II-1) and her affected cousin's mother (II-5) both carried a heterozygous *PRKN* mutation, c.856delT, and most of her relatives (II-2,6, III-5,9) carried the same heterozygous mutation. Her older brother (III-2) was found to be heterozygous for the mutation, c.1321T>C. The minor allele frequency (MAF) of c.1321T>C was recorded to be 0.00004 in gnomAD. It was classified as a disease-causing mutation (DM) in the Human Gene Mutation Database (HGMD, ID CM033800) and predicted to be “probably damaging” by Polyphen-2 and to “affect protein function” by SIFT. No allele frequency of c.856delT was recorded in gnomAD. Furthermore, it was neither recorded in HGMD nor reported by any other researchers. The prediction of MutationTaster on this mutation is that it is “disease causing.” The specific data are shown in Table 2.

**Table 2:** DNA sequencing results of the family.

**Gene symbol**	**DNA changes**	**MAF**	**HGMD**	**SIFT**	**Polyphen-2**	**MutationTaster**	**Carrier^1^**
*PRKN*	c.856delT	–	–	–	–	D	II-1,2,5,6; III-4,5,8,9
	c.1321T>C	0.00004 (gnomAD)	DM	D	D	D	III-2,4
*PLA2G6*	c.448G>A	0.00002 (gnomAD_exome)	–	T	D	D	II-1,2,6,7; III-2,3,4,5,6,9; IV-1
*TENM4*	c.6410C>T	0.00005 (gnomAD_exome)	–	T	P	D	III-3,4,8

*MAF, minor allele frequency; gnomAD, Genome Aggregation Database; HGMD, Human Gene Mutation Database; SIFT, Sorting Intolerant from Tolerant; Polyphen-2, Polymorphism Phenotyping v2; DM, disease-causing mutation; D (in SIFT), deleterious; D (in Polyphen-2), probably damaging; D (in MutationTaster), disease-causing; T, tolerated; P, possibly damaging*.

1 *The variant carriers detected in the family*.

Heterozygous variants c.448G>A (p.A2137V) of *PLA2G6* and c.6410C>T (p.E150K) of *TENM4* were detected in the proband, while c.6410C>T of *TENM4* was also found in the other affected family member. Details are shown in Table 2.

## Discussion

*PRKN*, the parkin RBR E3 ubiquitin protein ligase gene located in chromosome 6q25.2–q27.7 with 12 exons, was first reported as the genetic cause of autosomal recessive juvenile parkinsonism (ARJP) in 1998 in unrelated Japanese families (14, 15). Mutations of *PRKN*, mainly including exon rearrangements, deletions, or missense mutations, are also common in patients with EOPD in China (13, 16). Our team identified a novel heterozygous deletion and duplication of *PRKN* in a Han Chinese family with EOPD recently (17). American experts first detected compound heterozygous mutation Ex5 del/c.1321T>C of *PRKN* in patients with PD in 2002, and missense mutation c.1321T>C has been found in Asians with EOPD in recent years (18–21). However, the frameshift mutation c.856delT found in this study has never been reported before, nor has the compound heterozygous mutation c.1321T>C/c.856delT. Therefore, we conducted further protein function analysis *in silico* to predict the pathogenicity of these mutations.

The mutation c.856delT detected in this family results in amino acid alternation, in which the amino acid 286 is changed from serine to proline, which generates a new reading frame, stopping prematurely at codon 12 downstream (Figure 1C). The parkin protein is an E3 ubiquitin ligase implicated in mitophagy with 465 amino acids that functions in the ubiquitin–proteasome system, and the ubiquitin-like domain interacts with the RING1 domain in negatively regulating protein activity (9, 13, 22). Meanwhile, approximately 25% of all reported *PRKN* mutations have been found in the RING1 domain, as well as the mutation c.856delT found in our study (13). According to the prediction of MutationTaster, the novel frameshift mutation leads to amino acid sequence and splice site changes, and the protein features might be affected, which may ultimately result in the expression of truncated protein or becoming a target for nonsense-mediated mRNA decay. Accordingly, we infer that the mutation c.856delT of *PRKN* is deleterious. Furthermore, the missense mutation c.1321T>C is predicted to be deleterious by SIFT and Polyphen-2 and reported to be expected to affect ubiquitination (20). Therefore, it is reasonable to consider the mutation c.1321T>C as pathogenic, too.

For further cosegregation analysis, we performed direct sequencing of candidate genes on 13 members of this family. Mutation c.856delT was found in eight family members, while mutation c.1321T>C was found in two of them. All of the unaffected members carried a single heterozygous type. It can easily be determined that the mutation c.856delT is inherited from the proband's mother. Thus, we infer that the proband's father could be heterozygous for c.1321T>C, and her affected cousin's father (II-4), just like his siblings, carried a single heterozygous mutation, c.856delT. From the analysis above, these two candidate mutations are cosegregated from the disease phenotype in the family, and we believe that they are likely to be the genetic cause of PD in this family.

However, there were differences in the onset and manifestation of PD in the two patients of the family. The clinical features of *PRKN*-related PD were notable for the following points: early onset, mostly before 45 years old, responsive to levodopa, progressing slowly, commonly exhibiting dystonia, nearly half exhibiting hyperreflexia, no observed olfactory dysfunction, and limited suffering from dementia (9, 23–28). Patient III-8 appears to have typical symptoms of *PRKN*-related PD, such as early onset, slow progression, and no olfactory or cognitive dysfunction. In contrast, III-4 has a symmetrical tremor only in her legs, which has never extended to her upper limbs in the past 11 years. Moreover, she never exhibits rigidity and is more flexible than her affected cousin. According to the MDS clinical diagnostic criteria for PD mentioned previously (6), III-4 has one red flag (symmetrical parkinsonism, offset by one supported criteria) and one absolute exclusion criteria (parkinsonism limited to lower limbs for over 3 years). Principally, she would not be clinically diagnosed with PD for appearing incompatible with the diagnostic criteria. However, III-8 could be clinically diagnosed according to the criteria mentioned before, and she carries a homozygous frameshift mutation of *PRKN*, which is carried as heterozygous type by III-4. Based on our inferences above, the two affected members of the family could both be genetically diagnosed with PD.

Furthermore, ^18^F-DOPA PET/CT scan of III-4 showed reduced signaling in the bilateral striatum. Combining her family history, genetic examination results, and PET/CT imaging, she could be considered to have PD. However, the symmetrical reduced signaling in the ^18^F-DOPA PET/CT scan seems atypical among the images of patients with PD. Although there is a report suggesting that patients with *DJ1*-related PD exhibit early-onset levodopa-responsive symmetrical parkinsonism (29), to the best of our knowledge, there is no report of another patient with PD with an 11-year duration who exhibits symmetrical tremor exclusively limited to the lower limbs and no manifestation of rigidity. The patient appears incompatible with the MDS clinical diagnostic criteria, and although it may be an individual case, we, nonetheless, think it helps to enrich our understanding of PD diagnosis. Furthermore, the cause of the particular phenotype may also be genetic.

Additionally, we found that heterozygous variants c.448G>A (p.A2137V) of *PLA2G6* and c.6410C>T (p.E150K) of *TENM4* in the proband were predicted as “tolerated” by SIFT. Predictions on Polyphen reveal that the variant-locating sequences are relatively conserved, and the MAFs recorded in gnomAD are both lower than 0.01. Moreover, the variant c.6410C>T is also identified as a heterozygous type in the other affected family member. Studies show that some homozygous and compound heterozygous mutations of *PLA2G6* were found to be associated with EOPD in the Asian population (30–32). A homozygous missense mutation of *PLA2G6* was confirmed to contribute to autosomal recessive early-onset parkinsonism (AREP) in Han Chinese families without dystonia (33). It is also reported that a Chinese EOPD patient carrying a heterozygous mutation c.1321T>C of *PRKN* appeared to have symmetrical parkinsonism at onset (19). We boldly speculate that the special phenotype of the proband may be related to the two variants she carries, even though the other affected family member does not carry these mutations. However, whether or not these variants contribute to the symmetrical symptoms still requires further study. Some missense mutations of *TENM4* are confirmed to cause essential tremor in Caucasian populations; however, the relationship between *TENM4* and essential tremor in the Han Chinese population still needs further clarification (34–36). A recent study identifies *TENM4* as a novel candidate gene for a Han Chinese family with schizophrenia (37). The effect of the *TENM4* variant found in this study on the disease phenotype of the family still remains unclear.

Nevertheless, there are still some drawbacks in our research. We failed to obtain brain imaging data from the other patient of the family due to her trouble traveling, which might have been helpful in analyzing the cause of the difference in clinical features between the two patients. Furthermore, some members, including a patient's father, refused to participate in this study, which has an impact on pedigree analysis.

In conclusion, a novel homozygous frameshift mutation and a compound heterozygous mutation of *PRKN* were found in this EOPD family. The mutations c.856delT and c.1321T> C/c.856delT are considered pathogenic. Moreover, the proband's symptoms appeared incompatible with the MDS clinical diagnostic criteria. The differences in clinical phenotypes of the two patients in this family might be related to the slight difference in their genotypes. Further studies are required to clarify the pathogenicity of these mutations and to definitely understand their functional effects. In addition, we believe that clinical doctors need to consider whether it is necessary to improve the diagnostic criteria for PD and draw up targeted diagnostic criteria for familial hereditary PD.

## Data Availability Statement

The original contributions presented in the study are included in the article/Supplementary Materials, further inquiries can be directed to the corresponding author/s.

## Ethics Statement

The studies involving human participants were reviewed and approved by ethics committee of Nanjing First Hospital, Nanjing Medical University. The patients/participants provided their written informed consent to participate in this study. Written informed consent was obtained from the individual(s) for the publication of any potentially identifiable images or data included in this article.

## Author Contributions

YZ and YT conceptualized the study. CG and TH were responsible for the diagnosis and clinical evaluation. CG and ZY were in charge of the genetic analysis. CG, TH, and YT did the investigation. RC was responsible for the neuroimaging data and video collection. CG wrote the manuscript—original draft preparation. ZY, YT, and YZ wrote, reviewed, and edited the manuscript. CG and TH contributed equally to the study and should be considered as co-first authors. All authors have read and agreed to the published version of the manuscript.

## Conflict of Interest

The authors declare that the research was conducted in the absence of any commercial or financial relationships that could be construed as a potential conflict of interest.
